# Skin cancer detection through attention guided dual autoencoder approach with extreme learning machine

**DOI:** 10.1038/s41598-024-68749-1

**Published:** 2024-08-01

**Authors:** Ritesh Maurya, Satyajit Mahapatra, Malay Kishore Dutta, Vibhav Prakash Singh, Mohan Karnati, Geet Sahu, Nageshwar Nath Pandey

**Affiliations:** 1 Amity Centre for Artificial Intelligence, Amity University, Noida, India; 2 Department of Information and Communication Technology, Manipal Institute of Technology, Manipal Academy of Higher Education, Manipal, 576104 India; 3 Department of Computer Science and Engineering, Motilal Nehru National Institute of Technology Allahabad, Allahabad, India; 4 Computer Science and Engineering Department, National Institute of Technology Raipur, Chhattisgarh, 492010 India; 5 Department of Computer Science and Engineering, ITER, Siksha ‘O’ Anusandhan, Bhubaneswar, Odisha India

**Keywords:** Convolution neural networks, Skin lesion classification, Deep learning, Autoencoder, Extreme learning machine, Cancer, Computer science

## Abstract

Skin cancer is a lethal disease, and its early detection plays a pivotal role in preventing its spread to other body organs and tissues. Artificial Intelligence (AI)-based automated methods can play a significant role in its early detection. This study presents an AI-based novel approach, termed 'DualAutoELM' for the effective identification of various types of skin cancers. The proposed method leverages a network of autoencoders, comprising two distinct autoencoders: the spatial autoencoder and the FFT (Fast Fourier Transform)-autoencoder. The spatial-autoencoder specializes in learning spatial features within input lesion images whereas the FFT-autoencoder learns to capture textural and distinguishing frequency patterns within transformed input skin lesion images through the reconstruction process. The use of attention modules at various levels within the encoder part of these autoencoders significantly improves their discriminative feature learning capabilities. An Extreme Learning Machine (ELM) with a single layer of feedforward is trained to classify skin malignancies using the characteristics that were recovered from the bottleneck layers of these autoencoders. The 'HAM10000' and 'ISIC-2017' are two publicly available datasets used to thoroughly assess the suggested approach. The experimental findings demonstrate the accuracy and robustness of the proposed technique, with AUC, precision, and accuracy values for the 'HAM10000' dataset being 0.98, 97.68% and 97.66%, and for the 'ISIC-2017' dataset being 0.95, 86.75% and 86.68%, respectively. This study highlights the possibility of the suggested approach for accurate detection of skin cancer.

## Introduction

Skin cancer has exhibited a concerning global upsurge in recent years. In 2020, the data published by the World Cancer Research Fund International reported approximately 150,000 new cases of melanoma skin cancers worldwide, alongside a staggering 2–3 million cases of non-melanoma skin cancers, as disclosed by the World Health Organization in 2017. Data released by the American Cancer Society in 2018 underscores the critical importance of timely diagnosis, revealing a 5-year survival rate of 99% for melanoma when detected early. However, this rate drops to a mere 20% in cases where the disease has metastasized. Early signs of skin cancers elude easy identification and typically necessitate the expertise of a dermatologist^1^. Hence, there arises a need for AI-based tools in dermatological analysis, offering a second opinion and mitigating the misidentification rate^2,3^. The computer-aided detection (CAD) systems^4,5^ offers better accuracy and can be deployed at low cost^6,7^.

The intricate patterns present in the skin cancer lesion images pose a significant challenge in developing such CAD systems. These images have high inter-class similarity^8^. Even within the same class of skin lesions, images vary in color, texture and shape of a lesions^9^. These complexities make it a difficult task to accurately diagnose different types of skin cancer.

Traditional machine learning techniques have proven to be effective in categorising skin cancer^10^. However, these systems are prone to human error and subjected to the domain knowledge of human expert. Various feature extraction techniques have been utilized, including the Menzies approach^11^, the ABCD rule^12^, and the 7-point checklist^13^, for manual extraction of features. With the advancement in deep learning, networks such as autoencoders and convolutional neural networks (CNNs) have widely been used in many medical image recognition tasks^14–16^. These networks have been utilised for the tasks such as reconstruction, picture denoising, feature learning and object detection^17–19^. Despite the widespread use of deep learning-based methods, these methods suffer from the issues such as high computational cost, overfitting and the need for the large-annotated dataset^17^. The proposed method addresses the several key limitations of the conventional machine learning (ML)-based method such as unlike ML-based methods which rely on manual feature extraction, the proposed method relies on automatic extraction of spatial and frequency domain features utilising the reconstruction process in autoencoders. Other advancements such as the use of attention layer in the proposed method specifically used to increase the discriminability of the extracted features, makes the proposed method more advanced in comparison to the traditional ML-based approaches.

The proposed work suggests a "dual-autoencoder" network specifically designed for distinguishing various patterns in skin lesion images. This network comprises of two specialised autoencoders, with one specifically trained to extract spatial information such as shapes and structural characteristics from the input images. Whereas the second autoencoder is specialised in capturing textural differences and the differences in frequency patterns found in different categories of skin cancers, resulting in understanding more comprehensive representations in the frequency domain. Post removal of the decoder component, the feature representations that these autoencoders have learned are taken out of the trained bottleneck layers of these autoencoders. An attention mechanism has been included in the autoencoder network to improve the efficacy of the learnt feature representation by the suggested technique. This mechanism prioritizes the learning of pertinent patterns while effectively filtering out noisy or irrelevant elements within the input lesion images. Additionally, an efficient classification component, employing a single-layer, feed-forward Extreme Learning Machine has been employed. This choice of classifier not only expedites the training but also upholds a high level of accuracy. Altogether, the proposed method offers a robust and innovative solution for skin lesion classification with high diagnostic performance. The contributions of the proposed work can be summarised as follows:

1. An innovative architecture termed ‘DualAutoELM’ has been proposed. This architecture can extract highly discriminative spatial and frequency domain patterns present in an input skin lesion image.
2. The concept of multi-level attention has been integrated with the proposed method.
3. High classification accuracy of 97.66% and 86.68% has been obtained for the HAM10000 and ISIC2017 datasets which proves the generalisability of the proposed method.

The rest of the paper can be read as follows: Section "Related works" details related works. Section "Dataset used" details about the used dataset. Section "Proposed method" provides a detailed explanation of the methodology used in this work. Section "Experimental results" presents the experimental results, and the final section concludes the proposed work.

## Related works

In this section, a review of related works that are pertinent to the proposed research has been presented. Alenezi et al.^10^ introduced a method utilizing a wavelet-transform-based pre-trained ResNet101 model as a feature extractor, coupled with an ELM, for the classification of skin lesion images, achieving a categorisation performance of 95.75% with the HAM10000 dataset. Yin et al.^15^ innovatively combined unsupervised training of an autoencoder with supervised training of a classifier for autism detection, focusing on functional magnetic resonance imaging (fMRI) data. Cui et al.^20^ proposed an autoencoder-based approach for Maize disease identification, leveraging encoded features to train the fully connected layers in their model. Qian et al.^8^ introduced a method based on aggregating attention scores captured at different scales of skin lesion images using deep CNNs, incorporating class-specific loss weighting, and achieving a 91.6% classification accuracy with the HAM10000 dataset. Ding et al.^21^ presented a deep CNN with integrated attention mechanisms designed to generate class activation maps, enabling the network to focus on discriminative regions within input images, resulting in an AUC value of 0.922 with the ISIC2017 dataset.

In the domain of transfer learning, several notable works have made significant contributions to the field of skin cancer identification. Mendes and Krohling^22^ presented a method that combines deep and handcrafted features with patient records for skin cancer prediction using images captured through a smartphone, known as PAD-UFES-20. Agarwal et al.^23^ addressed the detection of face masks utilizing pre-trained deep CNNs in conjunction with a single-layer feed-forward ELM as a classifier. Toğaçar et al.^11^ introduced an approach that amalgamates an autoencoder, MobileNetV2, and spiking neural networks for the categorisation of 1800 benign and 1497 malignant tumour images obtained from the ISIC dataset, achieving an impressive classification accuracy of 95.27%. Priyadharshini et al.^24^ proposed a method for malignant skin lesion detection using Teaching–Learning-Based Optimization and an ELM, securing an F1-score of 91.64% in distinguishing benign from malignant skin lesions based on a dataset collected from Kaggle. In their research, Elaziz et al.^25^ effectively identified key features by employing the Artificial Rabbits optimisation method along with the MobileNetV3 model for feature extraction. In fact, when testing their approach on the HAM10000 dataset, it yielded an impressive classification accuracy of 88.71%.

In the most recent research on skin cancer detection, Gomathi et al.^16^ implemented cutting-edge techniques to achieve impressive results. By leveraging deep convolutional neural networks in tandem with the Bacterial Foraging Optimisation and Particle Swarm Optimisation algorithms, they were able to successfully extract features and classify skin lesions belonging to the miscellaneous category. The accuracy rate of their method on the HAM10000 dataset reached an impressive 96.46%. Similarly, Goceri^1^ tackled the challenge of classifying seven different types of skin malignancies by introducing a novel approach—the fully convolutional adaptive capsule neural network. With a detection accuracy of 95.24% on the same dataset, their solution proved to be highly effective, highlighting the potential of incorporating capsule networks in dermatological investigations. In their recent study, Tsai et al.^26^ proposed a promising approach for tackling data imbalance in skin cancer classification. By combining predictions from separate models into an ensemble, they utilized the power of Style-GAN to categorize seven distinct skin malignancies. Their innovative techniques serve as a benchmark for evaluating our proposed methodology and provide a comprehensive overview of different approaches to addressing challenging skin cancer classification tasks.

## Dataset used

**Figure 1:**
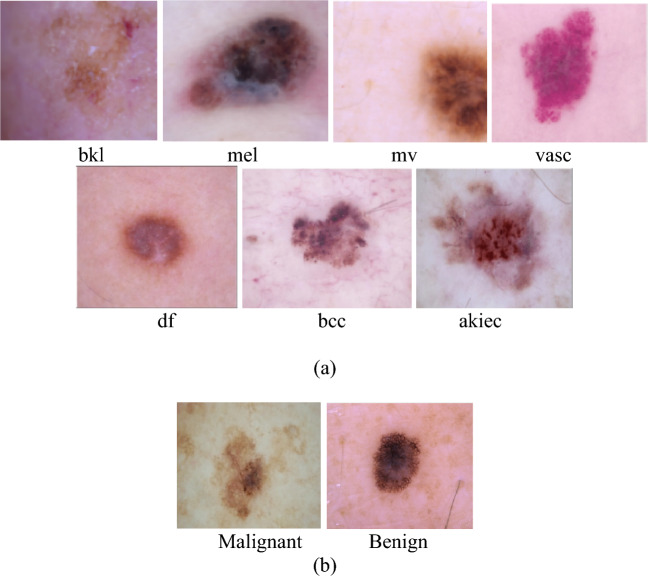
Sample images from each class for (**a**) HAM10000 and (**b**) ISIC-2017 dataset.

The proposed method was examined using the publicly available ISIC-2017^27^ and HAM10000^28^ datasets. These databases contain a variety of dermoscopic images of skin lesions. The training set of the ISIC-2017 dataset includes 2637 images, with an additional 660 images in the test set. In the same way, 10,015 dermoscopic pictures make up the HAM10000 dataset. ISIC-2017 dataset consists of two categories of skin lesion images: Benign and Malignant. HAM10000 dataset consists of seven skin lesion classes: Benign Keratosis (bkl), Melanocytic Nevi (nv), Basal Cell Carcinoma (bcc), Dermatofibroma (df), Melanoma(mel), Vascular lesions (vsc), and Actinic Keratosis and Intra-Epithelial Carcinoma (akiec). Some of the sample images from both datasets have been displayed in Fig. 1a,b.

## Proposed method

This paper presents a "DualAutoELM" model that utilises a mechanism based on a dual-autoencoder network with channel attention and an ELM classifier. With the capacity to recognise a wide range of skin cancers, this novel combination of components in the proposed method, optimises the feature extraction and classification process, providing a comprehensive solution for skin lesion classification. The working of the "DualAutoELM" method will be thoroughly explained in the section that follows along with its architecture, parts, and underlying reasoning that enables it to perform well for the present classification task.

### Pre-processing

The input images for the HAM10000 and ISIC2017 datasets were reshaped to have shapes of (28,28,3) and (64,64,3), respectively, as part of the pre-processing step. Since both datasets are imbalanced, therefore, the data imbalance has been removed by using random oversampling which randomly duplicates minority class samples in the training set with replacement. After oversampling images were normalised to bring the pixel values in the range 0–1. Other transformations such as one hot encoding, and data augmentation have also been performed.

### Autoencoders

Autoencoders are a class of neural networks used in various fields, including computer vision and data compression^20^. They aim to learn a compact representation of input data by encoding it into a lower-dimensional latent space and subsequently decoding it to reconstruct the original input. This process is mathematically represented as follows: given an input vector $x$, the encoding function is defined using Eq. (1).

1 $$f\left(x\right)=\sigma \left({W}_{e}x+{b}_{e}\right),$$

where ${W}_{e}$ and ${b}_{e}$ are the weight matrix and bias of the encoder respectively, and $\sigma$ is an activation function. The decoder is defined using Eq. (2):

2 $$g\left(f\left(x\right)\right)=\sigma \left({W}_{d}x+{b}_{d}\right),$$

where ${W}_{d}$ and ${b}_{d}$ are the weight matrix and bias of the decoder. Autoencoders are trained to minimize the reconstruction loss, typically using mean squared error (MSE) or binary cross-entropy loss, which is expressed a $L(x,g(f\left(x\right))$.

In this work, the concept of duality of autoencoders has been introduced which harnesses the reconstruction ability of autoencoders to learn skin lesion image representations from spatial and frequency domains.

### Channel attention module

**Figure 2:**
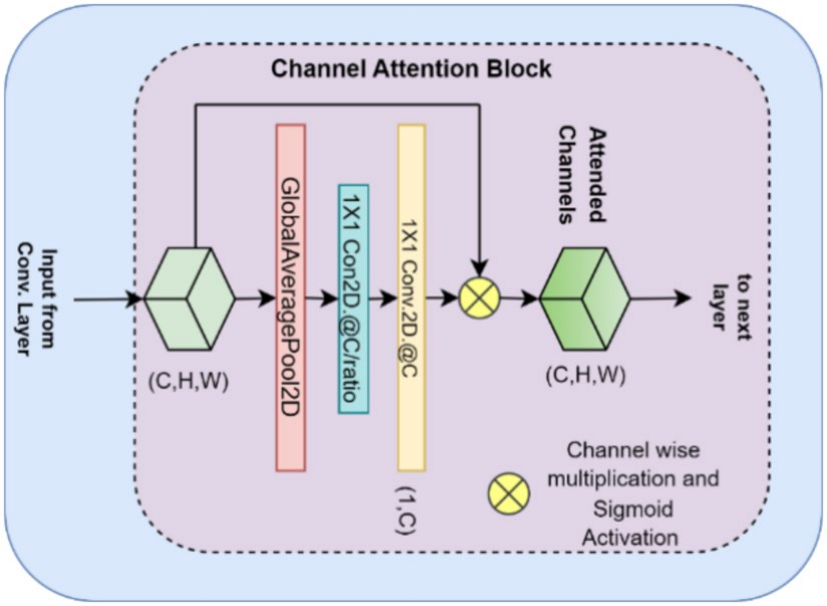
Channel attention mechanism.

The block diagram of the channel attention mechanism (CAM) used in the proposed method is shown in Fig. 2. The use of the CAM in the proposed Dual-autoencoder network enables the autoencoders to focus on the most informative channels while paying less attention to the less informative channels.

The overall functionality of the CAM used in the proposed work can be defined using Eqs. (3–6).

3 $$\text{GAP}\left(\text{x}\right)={\sum }_{i=1}^{i=H}{\sum }_{j=1}^{j=W}{x}_{i,j,C}$$

4 $${Y}_{1}=ReLU(Conv2D\left(GAP\left(x\right),\frac{c}{\text{r}},1\right))$$

5 $${Y}_{2}=\sigma \left(Conv2D\left({Y}_{1},C,1\right)\right)$$

6 $$\text{ChannelAttention}\left(\text{x}\right)=\text{x}\odot {\text{Y}}_{2}$$

$x$ is the input feature map and $x\in {\mathbb{R}}^{HXWXC}$. H (height) x W (width) x C (number of channels). ${x}_{i,j,c}$ is the value at spatial position $\left(i,j\right)$ in channel c. $\sigma$ denotes the sigmoid activation function. $\odot$ represents element-wise multiplication.$r,$ denotes compression ratio whose value has been set to 8, in this work. After GAP, a linear transformation is applied to the reduced tensor. The linear transformation consists of two convolutional layers. The first convolutional layer reduces the number of channels to $\frac{c}{\text{r}}$ with a 1 × 1 kernel size. A ReLU (Rectified Linear Unit) activation function is then applied to introduce non-linearity. The second linear transformation consists of another 1 × 1 convolutional layer, his layer brings the tensor back to its original number of channels (C), and a sigmoid activation function is applied. ${Y}_{2}$ is the output after the second linear transformation, and it contains values between 0 and 1, indicating the importance of each channel. Finally, the channel attention is applied to the input feature map $x$ by element-wise multiplication. This scales each channel by its importance score computed in the previous steps.

### Extreme learning machine

It is a fast supervised learning algorithm used for classification and regression tasks. It uses random initialisation of hidden layer weights and biases, distinguishing it from iterative optimization used in neural networks^10^. The hidden layer extracts features while the linear output layer learns the mapping to target values. The output of the ${i}^{th}$ hidden layer ${H}_{i}$ for the ${i}^{th}$ data point is computed using Eq. (7).

7 $${H}_{i}=\sigma \left({W}_{h}.{x}_{i}+{b}_{h}\right)$$

where $\sigma$ is the sigmoid activation function. ${W}_{h}$ represents the randomly initialized weights for the hidden layer. ${b}_{h}$ is a bias term for the hidden layer. The output layer weights, denoted as $\beta$, are learned by solving a linear regression problem. The goal is to find $\beta$ that minimizes the difference between the predicted values (hidden layer outputs) and the true target values. Thus, the linear regression problem can be formulated using Eq. (8).

8 $$T=H.\beta$$

where $T$ is the target values (true labels) for the training data. $H$ is the matrix of hidden layer outputs for all training samples. $\beta$ is the output weight matrix that needs to be learned. To find $\beta$, the Moore–Penrose pseudo-inverse technique has been used. The pseudo-inverse of *H*, denoted as *H* + , can be calculated using Eq. (9).Then, the output weights $\beta$ can be calculated using Eq. (10).

9 $${H}^{+}={(H}^{T}.{H)}^{-1}.{H}^{T}$$

10 $$\beta ={H}^{+}.T$$

This calculation yields the output weights that minimize the error between the hidden layer outputs and the true target values. Once these output weights are learned, they remain fixed for making predictions.

### Fast Fourier transform

The 2D Fast Fourier Transform (FFT2) is a mathematical operation used to transform an image, from the spatial to the frequency domain. It is a fundamental tool in signal processing and image analysis. Given an input array $X$ with $X\in {\mathbb{R}}^{HXW}$,representing a two-dimensional image array. Mathematically, the FFT2 operation can be represented using Eq. (11).

11 $$\text{Y}\left(\text{u},\text{ v}\right)={\sum }_{i=1}^{i=H}{\sum }_{j=1}^{j=W}{\text{X}\left(\text{i},\text{j}\right)\text{ e}}^{-\text{j}2\uppi \left( \left(\frac{{\text{u}}_{\text{i}}}{\text{H}}\right)*\text{ i }+ \left(\frac{{\text{v}}_{\text{j}}}{\text{W}}\right)*\text{ j}\right)}$$

where, $Y\left(u,v\right)$ represents the complex-valued frequency component at coordinates $\left(u,v\right)$ in the frequency domain. $X\left(i,j\right)$ is the value of the pixel at spatial coordinates $(i, j)$ in the input array. $H$ is the height of the input array. W is the width of the input array. ${u}_{i}$ and ${v}_{j}$ range from 0 to *(H-1)* and 0 to *(W-1)*, respectively, representing the spatial frequencies in the horizontal and vertical directions.

### Proposed dual auto-ELM method

**Figure 3:**
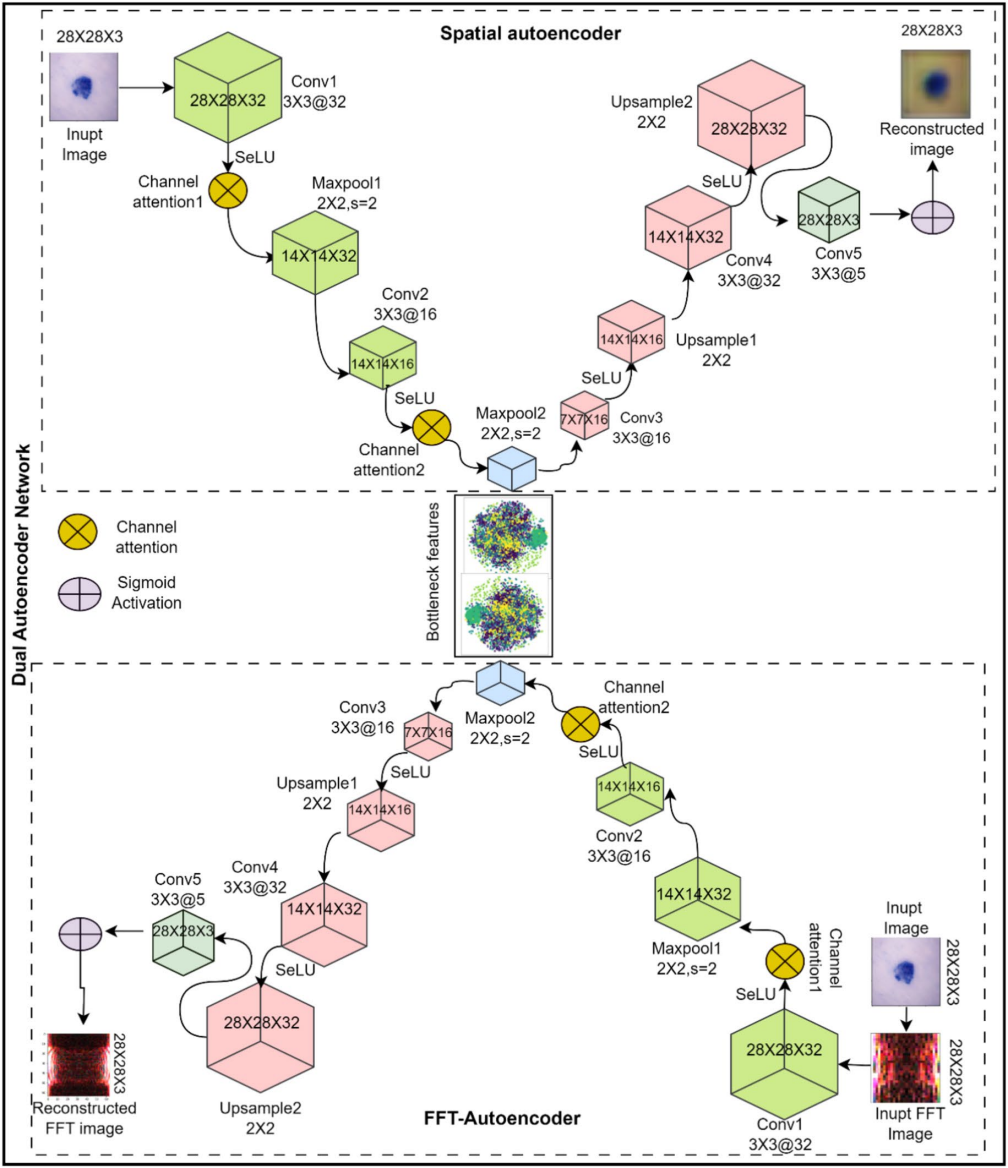
The proposed dual-autoencoder network.

**Figure 4:**
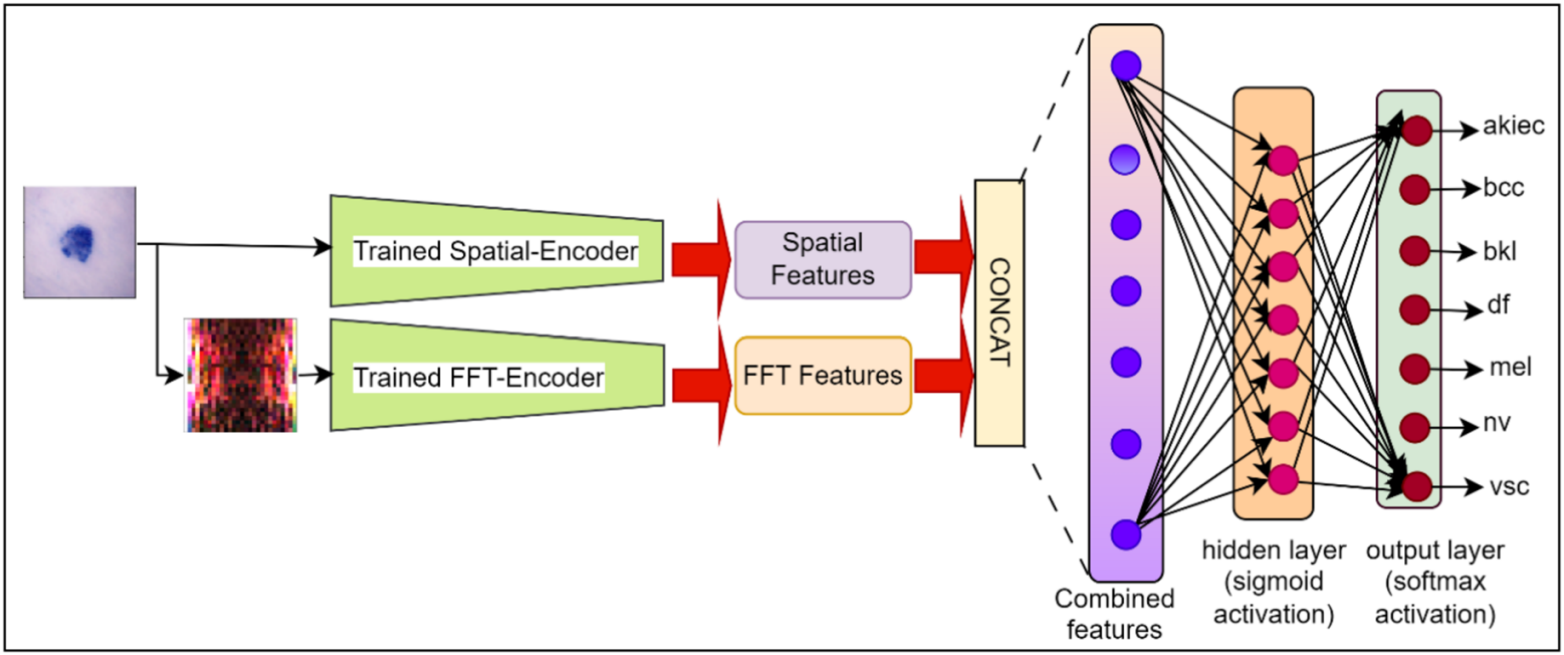
DualAutoELM model.

The detailed architectural overview of the proposed method has been presented using Figs. 3 and 4. As presented in Fig. 3, the proposed method consists of a network of two distinct autoencoders responsible for learning the representations from input lesion images; these autoencoders are termed ‘FFT-autoencoder’ and ‘spatial-autoencoder’. Skin lesion images often exhibit subtle textural variations, which can be better represented in the frequency domain. FFT-autoencoder, specialized in re-constructing Fast Fourier Transform (FFT)-transformed frequency domain representation of skin lesion images, thereby, capturing intricate frequency patterns present within the skin lesion images. During training, the FFT-Autoencoder model learns to reconstruct, discern, and extract the most informative frequency characteristics, allowing it to distinguish fine-grained differences in lesion images. Concurrently, the spatial-autoencoder is designed to capture spatial features present in the input lesion images such as shape, borders, and overall structure present in an input skin lesion image. As shown in Fig. 3, the integration of CAM, in Spatial-autoencoder and FFT-autoencoder helps in effectively filtering out the noise and emphasizing the most salient spatial and frequency domain features. This duality of autoencoders helps in the extraction of fine-grained representation from skin lesion images and makes the proposed model robust against recognising the different skin conditions.

The loss function used in both autoencoders is the Mean Squared Error (MSE) loss. The MSE loss evaluates the difference between the original input and the reconstructed output, promoting accurate reconstruction. Mathematically, it is defined using Eq. (12).

12 $$MSE-Loss\left(x,\widehat{x}\right)=\frac{1}{N}{\sum }_{i=1}^{N}{\left({x}_{i}-\widehat{{x}_{i}}\right)}^{2}$$

where $x$ is the original input,  $\widehat{x}$ is the reconstructed output and $N$ is the number of input elements in $x$. As shown in Fig. 3, the SeLU activation function^39^ has been used in place of ReLU within the convolutional layers of both autoencoders. The choice of SeLU activation is motivated by its self-normalizing properties, and its ability to mitigate the vanishing gradient problem, particularly results in better performance of the proposed model. The SeLU activation function can be mathematically expressed using Eq. (13).

13 $$SeLU\left(x\right)=\left\{\begin{array}{c}\lambda x, \quad x>0\\ \lambda \alpha {\left({e}^{x}-1\right)}, \quad x\le 0 \end{array}\right.$$

where, $x$ represents the input value. $\lambda$ is a scale parameter and $\alpha$ is a negative slope, whose value is set to 1.0507 and 1.67326 respectively. Let $X$ be the input feature map, $W$ be the convolutional kernel weights, $B$ be the bias, and $H$ be the output feature map. The application of SeLU activation function can be expressed using Eq. (14).

14 $$H=SeLU\left(X*W+B\right)$$

where, ∗ denotes the convolute and $H$ is the output feature map with SeLU activations.

#### Feature extraction from dual-autoencoder network and classification using extreme learning machine

The feature extraction and classification process have been illustrated in Fig. 4.

After unsupervised training of the FFT-autoencoder and Spatial-autoencoder, the next step involves feature extraction. During this phase, the decoder sections of both autoencoders, responsible for reconstructing the input, are excluded, leaving only the encoder section. These encoder sections of both autoencoders have learned to capture high-level, abstract features from the spatial and frequency domain representations of input lesion images during unsupervised training. The layer from which features are extracted from an encoder part is referred to as the "bottleneck layer", which serves as a compact representation of the input data. The feature representations obtained from the bottleneck layer of the Spatial-encoder and FFT-encoder are denoted as ${F}_{bottlenec{k}_{spatial}}$ *and* ${F}_{bottlenec{k}_{FFT}}$ respectively, and these are combined to form a combined feature representation denoted as ${F}_{bottleneck}$. ${F}_{bottleneck}$ retains valuable information about the underlying frequency domain patterns and spatial domain structures present within the skin lesion images, rendering it highly suitable for the skin cancer classification task at hand.

After feature extraction, the ELM classifier is employed for the subsequent classification. The combined feature representation ${F}_{bottleneck}$ serves as the input to the ELM classifier. ELM is trained to map these high-level features to the desired skin cancer class. Unlike traditional deep learning approaches that involve iterative optimization, ELM utilizes a fixed random projection in the hidden layer, resulting in significantly faster training. Mathematically, the feature extraction and classification process can be defined using Eqs. (15)–(18).

15 $${F}_{bottlenec{k}_{FFT}}={E}_{bottlenec{k}_{FFT}}\left(abs(FFT(X\right))), X\in {\mathbb{R}}^{HXWXC}$$

16 $${F}_{bottlenec{k}_{Spatial}}={E}_{bottlenec{k}_{spatial}}\left(X\right), X\in {\mathbb{R}}^{HXWXC}$$

17 $${F}_{bottleneck}={F}_{bottlenec{k}_{spatial}}\cup {F}_{bottlenec{k}_{FFT}}$$

18 $$\widehat{y}=softmax\left({W}_{out}.\upsigma \left({W}_{h}.{F}_{bottleneck}+{B}_{h}\right)+{B}_{out}\right), {F}_{bottleneck}\in {\mathbb{R}}^{M}$$

where $X$ is an input lesion image and $\widehat{y}$ is the prediction made by the ELM. The hidden layers weights and biases of an ELM denoted as ${W}_{h}$ and ${B}_{h}$, are randomly generated. ${W}_{out}$ and ${B}_{out}$ are the weights and biases from hidden layer to the output layer. The FFT function converts an input lesion image from spatial to the frequency domain and the *abs()* function calculates the magnitude of frequencies present in FFT-transformed lesion images.

In summary, our proposed dual-autoencoder-based network, incorporating a channel attention mechanism for channel-wise feature selection, and a SeLU activation for introducing non-linearity, utilizes the MSE loss for network optimization during training. The proposed architecture adaptively learns spatial and frequency domain features from an input skin lesion image. Subsequently, the combined representation obtained from bottleneck layers of the FFT-encoder and Spatial-encoder, is used for an efficient classification of skin lesion images using ELM.

## Experimental results

Different experimental details and results related to the proposed work have been presented in this section as follows:

### Experimental details

In this work, a novel approach for skin cancer categorisation based on a dual-autoencoder framework and an ELM has been proposed. All experiments were conducted on a computing platform equipped with an Nvidia P100 GPU, and 12 GB of RAM. The implementation of the proposed methodology is executed using the Keras 2.14.0 library with the Python programming language. The assessment of the proposed method involves evaluation metrics, including precision, recall, F1-score, Area Under the Curve (AUC), and accuracy.

To evaluate the effectiveness of our proposed approach, two distinct publicly available datasets, namely ISIC-2017 and HAM10000 have been used. The ISIC-2017 dataset was composed of 2637 images in the training set and 660 images in the test set. It encompasses 1440 skin lesion images in the benign class and 1197 skin lesion images in the malignant class. Conversely, the HAM10000 dataset encompasses 10,015 dermoscopic images. The datasets employed in this study exhibit class imbalances. To address this issue, a data augmentation technique has been used to alleviate the disparities in class distribution. The shape of the input image in the case of the ISIC-2017 dataset and the HAM10000 dataset was re-shaped to (64, 64, 3) and (28, 28, 3) respectively. To ensure a robust evaluation, a split ratio of 0.8:0.2 has been used. Post-data augmentation, 80% of the data was allocated to train the model, remaining 20% of the data was used to test the model.

### Data augmentation

Augmentation techniques including rotation (up to 20°), horizontal and vertical shifts (10%), horizontal flipping, and zooming (10%) have been used to enhance the ability of the proposed model to recognize skin lesions from different angles, locations, and scales.

### Hyperparameters

The ‘MSE’ loss function, the 'Adam' optimizer, and a batch size of 128 were used to train the autoencoders. The 'sigmoid' activation function was applied to the hidden layer of the Extreme Learning Machine (ELM) classifier with 64 neurons. 'Softmax' activation is used by the output layer of ELM classifier. From the training data, a validation set comprising 20% of the sample was chosen for model assessment. According to Alenezi et al. (2023), the hidden layer weights were initialised with random values and the output layer weights were computed using the Moore–Penrose Matrix Inverse function in ELM^10^.

### Results

The experimental results obtained from testing the recommended approaches on the ISIC-2017 and HAM10000 datasets are presented in this section. After the FFT-autoencoder and Spatial-autoencoder were trained, features were taken out of the "Maxpool2" layer of both the autoencoders. The Extreme Learning Machine (ELM) classifier is trained using this unified feature set, which is created by combining the extracted features. Because the suggested dual-autoencoder network's input picture shape varies for each of the two datasets, there will be differences in the output shapes at every layer of the two autoencoders. Table 1 displays the output form and number of parameters at each autoencoder layer for the HAM10000 and ISIC-2017 datasets. The bottleneck layer has been highlighted in bold in Table 1.

**Table 1:** Output shape and number of parameters for Spatial-autoencoder and FFT-autoencoders for HAM10000 and ISIC-2017 dataset.

	Layer name	Output shape (HAM10000)	Output shape (ISIC-2017)	#Params
Encoder	Input	[28,28,3]	[64,64,3]	0
	Conv1	[28,28,32]	[64,64,32]	896
	Channel_attention1	[28,28,32]	[64,64,32]	256
	Maxpool1	[14,14,32]	[32,32,32]	0
	Conv2	[14,14,16]	[32,32,16]	4624
	Channel_attention1	[14,14,16]	[32,32,16]	64
	**Maxpool2**	**[7,7,16]**	**[16,16,16]**	**0**
Decoder	Conv3	[7,7,16]	[16,16,16]	2320
	UpSample1	[14,14,16]	[32,32,16]	0
	Conv4	[14,14,32]	[32,32,32]	4640
	UpSample2	[28,28,32]	[64,42,32]	0
	Conv5	[28,28,3]	[64,64,3]	867
	Total parameters			13,667

As depicted in Table 2, the shape of the unified feature set given as an input to the ELM classifier for the HAM10000 and ISIC-2017 is (1,1568) and (1,8192), respectively. The corresponding details regarding the output shape and the number of parameters for the ELM classifier, configured with 64 hidden neurons, are presented in Table 2.

**Table 2:** Output shape and number of parameters for ELM for HAM10000 and ISIC-2017 dataset.

Layer name	HAM10000		ISIC-2017	
	Output shape	#Params	Output shape	#Params
Input	[1,1568]	0	[1,8192]	0
Hidden layer	[1,64]	100,416	[1,64]	524,352
Output layer	[1,7]	455	[1,2]	130
Total parameters	–	100,911	–	524,482

**Figure 5:**
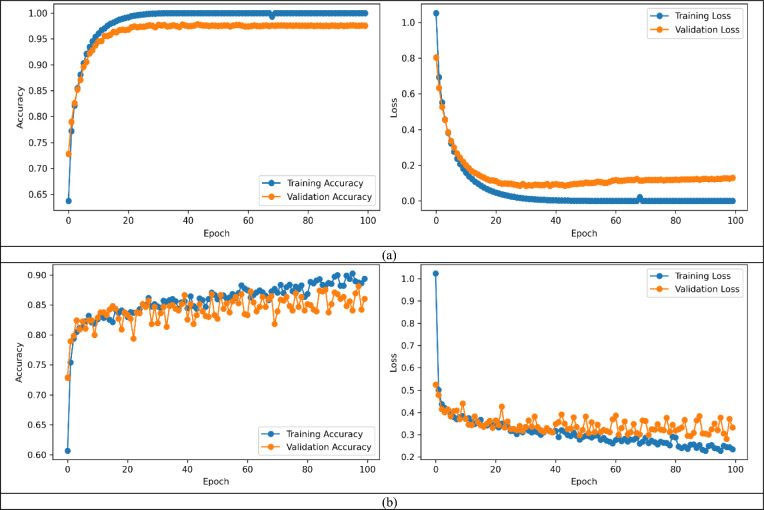
Training and Validation accuracy and loss curves for (**a**) the HAM10000 dataset and (**b**) the ISIC-2017 dataset.

Training and validation accuracy/loss curves for the ELM classifier for HAM10000 and ISIC-2017 datasets, have been displayed as Fig. 5a,b, respectively.

An examination of Fig. 5a,b reveals that no signs of overfitting or underfitting are present. This observation underscores the fact that when employing the combined feature set to train the proposed ELM classifier, an optimal fit is achieved.

**Figure 6:**
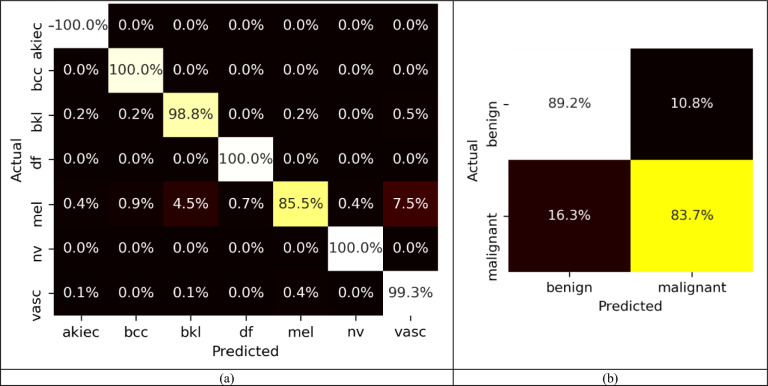
Confusion matrix for ELM classifier with (**a**) HAM10000 (**b**) ISIC-2017 dataset.

Figure 6a,b display the confusion matrices generated for both the HAM10000 and ISIC-2017 datasets. For the HAM10000 dataset, the ELM classifier exhibits remarkable classification performance with 100% accuracy achieved for the 'akiec,' 'bcc,' 'df,' and 'nv' classes. For ISIC-2017 dataset, the ELM classifier demonstrates superior performance in classifying 'benign' class samples compared to 'melanoma' class samples. Overall, the suggested method achieves an impressive average classification accuracy of 97.66% for the HAM10000 dataset and 86.68% for the ISIC-2017 dataset.

Performance assessment metrics such as precision, recall, and f1-score, are presented for each class, corresponding to the HAM10000 and ISIC-2017 datasets, have been presented in Tables 3 and 4 respectively.

**Table 3:** Value of different performance metrics for the HAM10000 dataset.

Class	Precision	Recall	F1-score	AUC
akiec	0.99	1.00	1.00	0.99
bcc	0.99	1.00	0.99	0.99
bkl	0.95	0.99	0.97	0.95
df	0.99	1.00	1.00	1.00
Mel	0.99	0.86	0.92	0.96
nv	1.00	1.00	1.00	1.00
vasc	0.93	0.99	0.96	0.96
Average	0.98	0.98	0.98	0.98

**Table 4:** Value of different performance metrics for the ISIC-2017 dataset.

Class	Precision	Recall	F1-score	AUC
Benign	0.87	0.89	0.88	0.95
Malignant	0.87	0.84	0.85	0.95
Average	0.87	0.87	0.87	0.95

**Figure 7:**
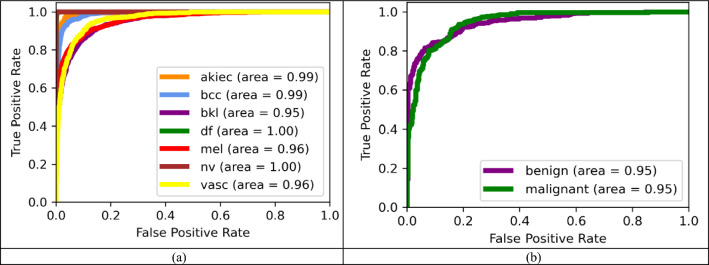
ROC curve for (**a**) HAM10000 and (**b**) ISIC-2017 dataset.

The average precision, recall, f1-score and AUC value of 0.98 were achieved for the HAM10000 dataset. For, ISIC-2017 dataset, the average precision, recall, and f1-score of 0.87, with an average AUC value of 0.95 has been obtained. ROC-AUC curves for each class present in both datasets have been shown in Fig. 7.

#### Ablation study

An ablation study has been conducted to analyse the impact of different components of the DualAutoELM method on its performance. The use of ELM in place of multi-layer perceptron (MLP) has been rationalising by observing the performance of the proposed method by replacing the ELM with two layers of MLP consisting of two hidden layers with 64 and 128 neurons and this model is termed DualAutoMLP. The comparative analysis of the proposed ‘DualAutoELM’ method and the ‘Dual-AutoMLP’ method, with and without augmentation, has been presented in Table 5.

**Table 5:** Comparison of %Accuracy obtained by DualAutoELM and DualAutoMLP model with or without data augmentation.

Dataset	Data augmentation	DualAutoELM	DualAutoMLP
ISIC-2017	No	85.05	83.43
	Yes	86.68	84.55
HAM10000	No	94.14	93.09
	Yes	97.66	95.01

Analysing the data presented in Table 5 justifies the use of ELM and data augmentation in the methodology proposed in this work. To justify the use of Dual-autoencoder-based architecture the performance of the ELM classifier with the features extracted from the different components of the Dual-autoencoder has been analysed and presented in Table 6.

**Table 6:** Performance of ELM classifier with the features extracted from spatial-encoder, FFT-encoder and dual-autoencoder for HAM10000 and ISIC-2017 dataset.

Features extracted from the bottleneck layer of	%Accuracy (HAM10000)	%Accuracy (ISIC-2017)
Spatial Autoencoder	94.27	85.11
FFT-Autoencoder	93.76	83.09
Combined feature (spatial Autoencoder + FFT-Autoencoder)	97.66	86.68

It can be observed from Table 6 that the suggested method has outperformed when the combined features have been used to train the ELM classifier. To justify the use of the SeLU activation function, the ReLU activation function has been replaced with the SeLU activation function in the proposed DualAutoELM and the corresponding performance of the DualAutoELM method has been reported in Table 7.

**Table 7:** Performance comparison with different activation functions for the proposed DualAutoELM model.

Activation	Dataset	
	HAM10000	ISIC-2017
ReLU	95.38	84.23
SeLU	97.66	86.68

As presented in Table 7, the performance of the proposed framework with the SeLU activation function has been justified over the use of the ReLU activation function.

Thus, this ablation study justifies the use of different components in the proposed framework. The next section makes a visual analysis of the proposed model to improve its interpretability.

#### Visualization

**Figure 8:**
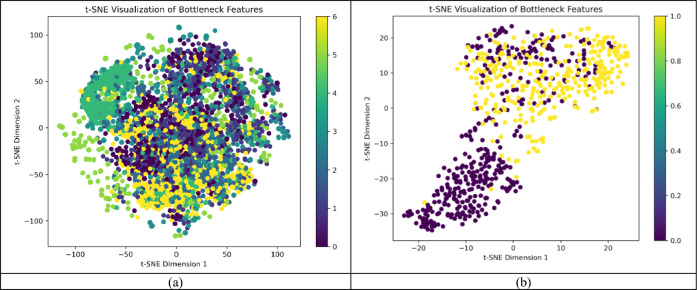
t-SNE visualisation of the bottleneck features for (**a**) HAM1000 and (**b**) ISIC-2017 dataset.

With the help of visualisation, the working of the DualAutoELM method has been analysed. To represent the discriminability of the high-dimensional features extracted from the bottleneck layer t-SNE plot has been plotted to represent high-dimensional features in two-dimensional space as shown in Fig. 8.

**Figure 9:**
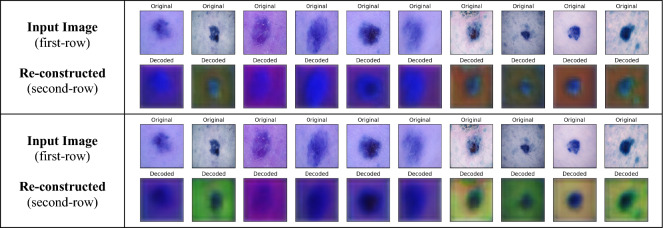
Input image and the image re-reconstructed by the spatial-autoencoder.

The reconstructed images with the spatial-autoencoder have been analysed to understand the quality of representation of input images in latent space. Figure 9 shows the input image and the image reconstructed by the spatial autoencoder. It can be analysed from Fig. 9 that the quality of re-constructed images is not of high quality though the autoencoder has been successful in capturing the spatial properties of an input lesion image.

**Figure 10:**
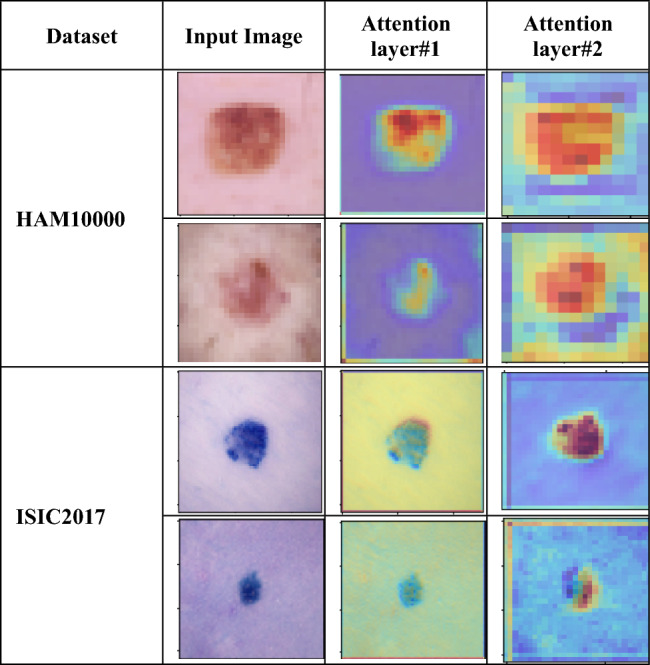
Visualisation of heat maps for the attention layers used in the DualAutoELM framework.

To visualise the region of focus of attention layers used in the autoencoder, heat maps obtained from individual attention layer has been visualised as shown in Fig. 10.

It can be analysed from Fig. 10 that the channel attention layers used in the proposed dual-autoencoder network focus on the region where the lesion is found to be present. The performance of the DualAutoELM method has also been compared with the other state-of-the-art method proposed by the other researchers for skin lesion classification, in recent years.

#### Comparison with other related works

The comparison of the experimental results obtained with the DualAutoELM method with other related works has also been made as presented in Table 8.

**Table 8:** Comparison with other related works.

References	Dataset	Method	Precision	Recall	AUC	Accuracy
^25^	HAM10000	Features extracted from the pre-trained MobileNetV3 were optimised using Artificial Rabbits optimisation algorithm based on Gaussian mutation and crossover operator	0.9002	0.8965	–	88.71
^16^	HAM10000	UNet was used to segment the skin lesion image and the features were extracted from the segmented images using hybrid optimisation algorithm by combining Bacterial Foraging Optimization (BFO) and Particle Swarm Optimization for classification with the deep CNN	96.02	95.37	0.98	96.46
^3^	HAM10000	A Deep Belief Network has been proposed for the classification of skin lesion using improved Electromagnetic Field Optimization algorithm	94.99	–	–	85.99
^1^	HAM10000	Convolutional capsule layers were added in the Capsule Neural network for the multi-class classification of skin lesions	95.37	–	–	95.24
^10^	HAM10000	Wavelet transform and ResNet101 model has been used to extract the discriminatory features using wavelet transformation, pooling, and normalization for skin lesion classification	95.84	–	–	95.73
^29^	HAM10000	High dimension contrast transforms for segmentation of skin lesions and then the features extracted from the pre-trained DenseNet201 using segmented images were classified using Extreme Learning Machine	–	–	–	88.39
^30^	HAM10000	They have combined discrete wavelet transform with CNN architecture for the classification of skin cancerous lesion images	94.00	91.00	–	94.00
^31^	HAM10000	They have proposed an ensemble of five different CNNs combined using weighted average ensemble method	–	–	96.00	–
^32^	ISIC2017	Features were extracted from the segmented lesion images using Region Average Pooling method and classification using a Linear Classifier	60.7	–	0.842	83.00
^33^	ISIC2017	Full resolution convolutional network for segmentation of lesion from the dermoscopic images and the classification of segmented images using deep CNNs	–	75.33		81.57
^34^	ISIC2017	Handcrafted and deep features were extracted from the segmented lesion image and classified using support vector machine classifier	–	–	–	85.3
^35^	ISIC2017	Pre-trained NASNetMobile model has been fine-tuned for skin lesion classification task	81.77	–	–	82.00
^36^	ISIC2017	Color, shape and texture features were extracted from the skin lesion image and classified using k-Nearest Neighbour classifier	–	80.00	0.68	–
^37^	ISIC2017	Two dimensional Fourier transformed image is passed to a customized CNN for feature extraction	–	–	0.93	83.00
^38^	Kaggle dataset	Deep features have been concatenated and K-best features were selected and classified using XGBoost classifier	85.5	–	–	85.91
Proposed method	HAM10000 ISIC-2017	Dual autoencoder networks have been used one for the extraction of the spatial features and other for the extraction of frequency domain features; the multi-level attention has been integrated in the encoder part; the combined features were classified using Extreme Learning Machine	97.68 86.75	97.59 86.62	0.98 0.95	97.66 86.68

It can be analysed from Table 8 that the proposed method outperformed the other recent related works on several performance metrics. Therefore, the proposed method stands out due to its robustness and high classification performance for skin cancer classification tasks.

## Conclusion

This work proposes a dual-autoencoder-based approach for learning the feature representation for multiple types of skin cancer identification and utilises extreme learning machines for classifying them into relevant categories. The capability of the DualAutoELM method to learn feature representation from spatial and frequency domain automatically makes it interesting and ELM plays a significant role in decreasing the computational complexity of the proposed method. The proposed method outperformed other works when tested with the HAM10000 and ISIC-2017 datasets. It has achieved classification accuracy of 97.66% and 86.68% with the HAM10000 and ISIC-2017 dataset respectively which makes it robust and efficient in identifying multiple types of skin cancers using the proposed method.

Despite the high classification performance of the proposed method certain limitations still need to be acknowledged. Though the proposed method has been tested on two diverse datasets, i.e., HAM10000 and ISIC2017, still it would be not sufficiently comprehensive testing strategy considering the variability among the skin lesion images captured in real-world clinical settings. Therefore, clinical validation of the proposed method in real-world setting needs to be performed. Additionally, the proposed model does not consider the integration of patient metadata such as age, sex and medical history which can significantly improve the performance of the proposed model. The future direction of the proposed research may be as follows:

1. Diversity of the dataset will be increased by including the skin lesion images captured in real-world clinical setting, so that generalizability and the robustness of the model can be evaluated in a more comprehensive manner.
2. Important patient’s meta-data such as age, sex and medical history will be integrated with the proposed model for accurate predictability of the skin cancer.
3. A mobile application will be interfaced with the trained DualAutoELM model.

By addressing these limitations and exploring the possibilities in future direction of the present work, the potential of the proposed method in significantly impacting the early detection of and diagnosis of skin cancer can be further realized.

## Data Availability

The datasets that support the findings of this study are available on ISIC archive (https://isic-archive.com/) and the Harvard Dataverse repository (https://dataverse.harvard.edu/dataset.xhtml?persistentId=doi:10.7910/DVN/DBW86T).
